# Extracellular Matrix Structure and Composition in the Early Four-Chambered Embryonic Heart

**DOI:** 10.3390/cells9020285

**Published:** 2020-01-24

**Authors:** Quentin Jallerat, Adam W. Feinberg

**Affiliations:** 1 Department of Biomedical Engineering, Carnegie Mellon University, Pittsburgh, PA 15213, USA; qjallerat@gmail.com; 2 Department of Materials Science & Engineering, Carnegie Mellon University, Pittsburgh, PA 15213, USA

**Keywords:** fibronectin, laminin, collagen type IV, heart development, cardiogenesis, myocardium, extracellular matrix, confocal microscopy

## Abstract

During embryonic development, the heart undergoes complex morphogenesis from a liner tube into the four chambers consisting of ventricles, atria and valves. At the same time, the cardiomyocytes compact into a dense, aligned, and highly vascularized myocardium. The extracellular matrix (ECM) is known to play an important role in this process but understanding of the expression and organization remains incomplete. Here, we performed 3D confocal imaging of ECM in the left ventricle and whole heart of embryonic chick from stages Hamburger-Hamilton 28–35 (days 5–9) as an accessible model of heart formation. First, we observed the formation of a fibronectin-rich, capillary-like networks in the myocardium between day 5 and day 9 of development. Then, we focused on day 5 prior to vascularization to determine the relative expression of fibronectin, laminin, and collagen type IV. Cardiomyocytes were found to uniaxially align prior to vascularization and, while the epicardium contained all ECM components, laminin was reduced, and collagen type IV was largely absent. Quantification of fibronectin revealed highly aligned fibers with a mean diameter of ~500 nm and interfiber spacing of ~3 µm. These structural parameters (volume, spacing, fiber diameter, length, and orientation) provide a quantitative framework to describe the organization of the embryonic ECM.

## 1. Introduction

The study of embryonic development has spanned centuries and has illuminated many of the mechanisms that guide form and function in the human body. In this context, the heart is unique, as it is the first organ to form and, once it begins to beat, it continues to do so throughout the major transformations of the embryo. Cardiac morphogenesis is a good example of this complex process, where the early contractile linear heart tube loops around to create distinctive chambers (ventricles and atria) that mature and septate. Advances in microscopy and cell lineage tracking have led to a better understanding of the mechanical aspects of cardiac formation [1] as well as the origin of cell populations making up the first and second heart fields [2,3], coronary vasculature [4,5,6] and the conduction system [7]. Researchers have also been able to put together stage by stage guides for different species to study similarities and differences [8]. However, the role of the extracellular matrix (ECM), which provides mechanical support and is key to many cell signaling processes, has not been fully studied during this process. The fibronectin-rich ECM appears to be critical to heart formation, with distinct fibronectin fiber morphology associated with each stage of cardiac looping [9]. Knock-out studies of different ECM proteins have also shown that removing laminin, fibronectin, or collagen type IV is embryonically lethal [10]. More studies are needed though to quantitatively characterize the composition and structure of the cardiac ECM at the microscale.

A number of tools and techniques have been used to study the cardiac ECM in order to quantify structure and composition. For example, Ogle and co-workers used immunohistochemistry to analyze the expression of elastin, fibronectin, collagen type I, and collagen type IV in the epicardium, myocardium and endocardium of the embryonic and adult mouse heart [11]. They showed changes in the relative concentration of each ECM protein in the myocardium during development, however, absolute composition and 3D architecture of the ECM were difficult to quantify in the sectioned, paraffin-embedded samples. To determine ECM composition in more detail, Williams et al. used mass spectrometry and found that fibronectin, collagen type V, and fibrillin are the main components of the fetal cardiac ECM, while laminin and collagen type I are the main components of the adult cardiac ECM [12]. These major differences between fetal and adult cardiac ECM highlight the potential role proteins such as fibronectin may play during cardiac morphogenesis. Furthermore, it indicates that studying the adult heart cannot be a substitute for understanding the ECM and morphogenesis of the embryonic heart. The limitation of mass spectrometry on the whole heart is that information regarding the spatial distribution and microstructure of the ECM is typically absent. To further understand the role of the ECM in cardiac development, we need to be able to describe the ECM at the microscale in terms of both structure and composition.

In this study, we used whole-mount confocal microscopy to characterize the structure and composition of ECM components in the developing heart with a focus on fibronectin. The chick embryo served as a model system because, at early time points, it is similar to the human embryo and provides easy access to the heart throughout development (stages Hamburger-Hamilton (HH) 27–35) [13]. This range was selected because it is the earliest stage at which highly-aligned layers of myocardium have formed in the ventricular wall, and the vasculature including capillaries begin to develop. We implemented an integrated fixation, immunofluorescent staining, optical clearing, confocal microscopy, and image analysis process to visualize and analyze fibronectin, laminin and collagen type IV. Finally, we adapted sample clearing protocols to image the whole heart. With a focus on the epicardium and myocardium, we identified distinct changes in tissue structure, ECM composition and ECM organization throughout these early time points. This study also demonstrates the use of advanced imaging to obtain quantitative information about the microstructure of the ECM. This type of information will improve understanding of cardiogenesis and also provides a quantitative template of ECM architecture for future applications such as cardiac tissue engineering for disease modeling and regenerative medicine.

## 2. Materials and Methods

### 2.1. Chick Embryonic Heart Dissection

**Figure 1:**
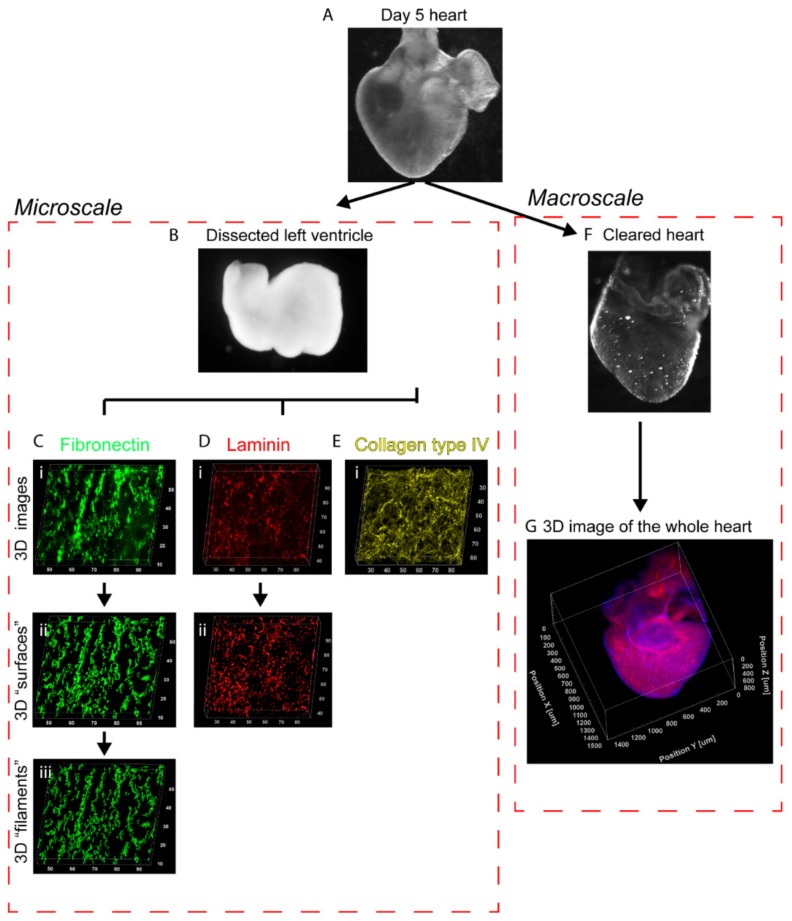
Schematic of the process to image and analyze the embryonic myocardium at the microscale and macroscale. (**A**) Chick embryonic hearts were dissected, fixed, and stained at different stages of development. (**B**) To study the extracellular matrix (ECM) at the microscale, the left ventricle was dissected and opened to lay flat. (**C**–**E**, i) High-resolution 3D images of the fibronectin, laminin, and collagen type IV ECM were obtained by confocal microscopy. (**C**,**D**, ii) The fibronectin and laminin images were segmented to create 3D surface objects to characterize the volume and spacing. (**C**, iii) The fibronectin images were further processed to create 3D filaments objects used to measure fiber diameter, length and orientation. (**F**) Hearts were also whole mounted in a 1:2 solution of benzyl alcohol to benzyl benzoate (BABB), a refractive index matching solution to clear the tissue. (**G**) 3D images of the whole heart, showing detailed trabeculae, were obtained using a macrozoom confocal microscope. Scale is in micrometers.

White Leghorn eggs were incubated at 37 °C with 50% humidity. On the day of dissection, embryos were explanted and put in a saline solution consisting of 1X PBS with calcium and magnesium (Sigma-Aldrich, St. Louis, MO, USA), buffered with 10 mM HEPES (Sigma-Aldrich). Embryos were then compared to the reference stages from Hamburger and Hamilton [13] to select a population with homogenous developmental stage: stage HH 28 for day 5, stage HH 31 for day 7, and stage HH 35 for day 9. Whole hearts were carefully dissected, keeping most of the outflow track (Figure 1A). For high-resolution imaging, the atria, outflow track, and right ventricle were removed. The left ventricles were then cut at the base in order to be flat for imaging (Figure 1B). This made a wide area available for high-resolution imaging as the high curvature of the embryonic heart would otherwise be an obstacle for the use of high magnification objectives with short working distance.

### 2.2. Fixation, Immunofluorescent Staining and Clearing

Whole hearts and isolated left ventricles were fixed for 15 min in 4% formaldehyde, then incubated for 2 h at 37 °C in PBS with 0.1% Triton X-100 (Sigma-Aldrich) and 5% goat serum for permeabilization and blocking. Hearts were then immunofluorescently stained for fibronectin, laminin and/or collagen type IV (Figure 1C–E). All incubations for staining were carried out overnight at 4 °C in 1X PBS with 5% goat serum or 10% bovine serum albumin. During the primary stain, either mouse anti-chicken fibronectin (Sigma-Aldrich, #F6140), rabbit anti-COL4A3BP (Sigma-Aldrich, #AV52418), rabbit anti-laminin (Sigma-Aldrich, #L9393), or mouse anti-sarcomeric α-actinin (Sigma-Aldrich, #A7811) were used at a 1:100 dilution, in addition to 3:100 Alexa Fluor 633-conjugated phalloidin (ThermoFisher, Waltham, MA, USA,#A22284) to stain F-actin. During the secondary incubation, goat anti-mouse antibodies conjugated to Alexa Fluor 546 (ThermoFisher, #A11030) and/or goat anti-rabbit antibodies conjugated to Alexa Fluor 488 (ThermoFisher, #A11008) were used at a 1:100 dilution. After each staining step, the samples were washed in 1X PBS (Sigma-Aldrich) three times for 30 min for each wash. One drop of NucBlue (ThermoFisher, #R37606) per mL was added during the second washing step after secondary staining to stain the nuclei. Finally, the samples were fixed post-stain for 1 h in 1X PBS with 4% formaldehyde. After three final rinses in 1X PBS, the whole hearts were cleared, and the flattened left ventricles were mounted directly in 1X PBS.

To clear whole hearts while preserving the F-actin staining, we followed a method by Kim et al. (Figure 1F) [14]. Stained whole hearts were washed in solutions of increasing concentration of isopropanol to 1X PBS (25%, 50%, 75%, 100%), and were then left to fully dehydrate in fresh isopropanol for 30 min. Isopropanol was replaced by BABB (1:2 solution of benzyl alcohol to benzyl benzoate, Sigma-Aldrich), which has a refractive index of 1.54 matching cardiac tissue. After 1 h to equilibrate in BABB, the samples were nearly transparent under the naked eye. Samples were then mounted with fresh BABB in a chamber made with a Fastwell (Electron Microscopy Sciences, Hatfield, PA, USA, # 70333-32) silicone ring between a microscope slide and a N1.5 glass coverslip.

### 2.3. High-Resolution Confocal Microscopy

Cleared whole hearts were imaged using a Nikon AZ-C2 macro zoom confocal (Nikon Instruments, Melville, NY, USA) with a 5× objective (NA = 0.4) (Figure 1F). For dissected left ventricles, in BABB or PBS, an X mark was bleached in the outflow track by scanning orthogonal lines with the 488 laser (Nikon Instruments) at minimum scan speed with 20–30% laser power for approximately 15 sec. A tiled z-stack was acquired with the same objective. This allowed us to keep track of the location of each high-resolution z-stack relative to the sample to make sure that only the left ventricle was imaged. Then 3D images were obtained using a high-resolution 63× oil objective (NA = 1.4) on a Zeiss LSM700 confocal microscope (Carl Zeiss Microscopy, White Plains, NY, USA). Samples were mounted in PBS but imaged with an oil objective, which introduced a strong mismatch in refractive indices, leading to spherical aberration and dilation of the *z*-axis [15]. We compensated for it by using the “refractive index correction” function of the Zeiss Zen 2010 software (Carl Zeiss Microscopy), which corrects spherical aberration using the ratio of the refractive indices of the medium of the sample (n = 1.33 for PBS) and of the immersion medium of the objective (n’ = 1.518 for oil), $R=\frac{n}{n\prime }=0.88$. We set the laser power and gain to increase through the depth of the myocardium to maintain appropriate signal intensity in each slice of the final 3D image stack.

### 2.4. Quantitative Analysis of 3D Image Z-Stacks of Laminin and Fibronectin

The 3D images from all samples were deconvolved with Autoquant X3. First, we cropped an area of the myocardium just below the basement membrane of the epicardium. For both laminin and fibronectin, we used the “surface” tool in Imaris 8.2 (Oxford Instruments, Concord, MA, USA) to segment the signal using the following parameters: local contrast threshold = 9, sphere diameter = 1 µm, smoothing = 0.1 µm. We filtered out elements that were smaller than 1 µm^3^ and sorted the “surfaces” according to their volume.

To measure spacing of the fibronectin and laminin “surfaces” created by Imaris, we used the “Distance to Surface” MATLAB Xtension (MathWorks, Natick, MA, USA) to create new channels where the pixels outside of the “surfaces” have values equal to the minimal distance to the ECM “surface”. The “Distance to” channels were further processed using the “surface” tool (local contrast threshold = 2, no smoothing, sphere diameter = 0.1 µm) to segment and mask the local maxima of the signal. The resulting channel “Local Maxima of Distance to ECM” shows a network of sheets that pass exactly between ECM “surfaces”: each voxel of this channel is equidistant to the closest ECM “surfaces” and the intensity is equal to the shortest distance to these surfaces. If the shortest path is drawn between two adjacent ECM “surfaces”, it will intersect the “local maxima of distance to ECM” orthogonally and in its center. The intensity of the “local maxima of distance to ECM” at the point of intersection will be exactly half of the length of the shortest path between ECM “surfaces”. The channel representing the “Local Maxima of Distance to ECM” were then analyzed with ImageJ to produce histograms with set bins for each 3D z-stack.

For fibronectin only, the “surfaces” were used to mask the original fibronectin signal and create a channel without background. We then used the “Filament Tracker” tool to segment the fibronectin signal into fibers with the following parameters selected: absolute intensity threshold, no preprocessing, and branch length to trunk radius ratio = 2. We removed loops than were shorter than 1 µm (those are often artifacts of the algorithm filling up larger volume of the signal with a “ball” of fibers). This analysis resulted in the fiber diameter and length. Finally, fibers longer than 1 µm were selected and their orientation angles relative to the X axis were used to create a histogram for each sample.

## 3. Results

### 3.1. Fibronectin Is Associated with the Emergence of Cappilaries in the Left Ventricle Myocardium

**Figure 2:**
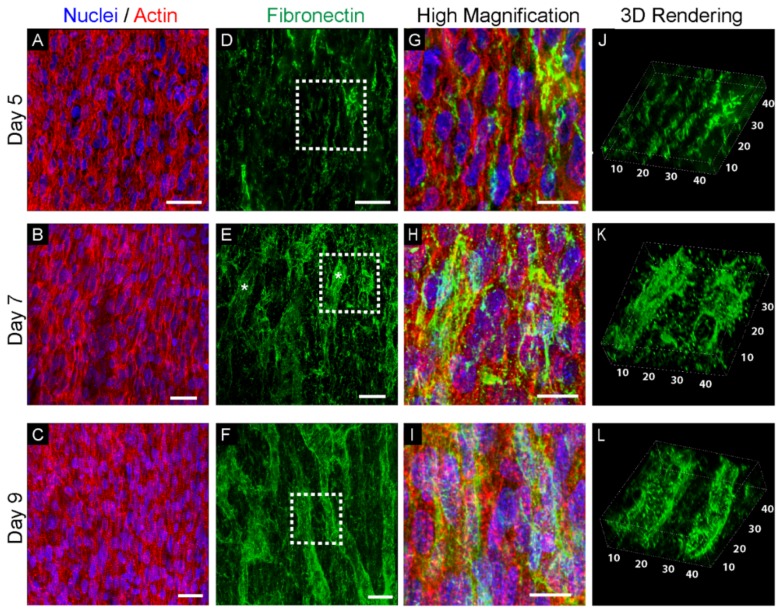
Imaging of the myocardium of the left ventricle at days 5, 7, and 9 of development. (**A**–**C**) Actin staining of the aligned cardiomyocyte cytoskeleton at days 5, 7, and 9 (red = actin, blue = nuclei). (**D**–**F**) Images of the fibronectin matrix (green) shows (**D**) very thin fibronectin fibers at day 5, (**E**) emergence of some larger bundles (marked by white stars) around preliminary capillary lumens at day 7, and (**F**) an interconnected network of fibronectin-positive tubes at day 9. (**G**–**I**) Magnified images of the areas marked by dashed white boxes in (**D**–**F**) show how the fibronectin matrix is organized around the cardiomyocytes. (**G**) At day 5, small fibronectin fibers are regularly located between cardiomyocytes, following the main direction of alignment. (**H**,**I**) At day 7 and day 9, the large bungles of fibronectin around the developing capillaries are also following the main cardiomyocyte orientation. (**J**–**L**) 3D rendering of the fibronectin shows: (**J**) at day 5, very small filaments with a main direction of alignment; (**K**) at day 7, the formation of much longer fibronectin structures with what appears to be poorly defined lumens; (**L**) at day 9, fibronectin highlighting what appears to be well-aligned capillaries. Scale bars are 20 µm (**A**–**F**) and 10 µm (**G**–**I**).

We fixed and stained hearts for fibronectin at days 5, 7, and 9 of development in order to visualize the emergence and timing of capillaries in the aligned myocardium. At all of the time points, the cardiomyocytes in the compacted layers of the myocardium were highly aligned as revealed by staining of the F-actin positive myofibrils (Figure 2A–C). However, there did appear to be an increase in the linearity of the myofibrils and the emergence of clear striations from day 5 to day 9. In contrast, the fibronectin showed distinct and drastic changes over time associated with the emergence of capillaries. At day 5, fibronectin formed small bundles of fibers interspaced between cardiomyocytes, following the main direction of alignment (Figure 2D). At day 7, structures began to appear, suggesting the creation of capillary-like tubes that had a fibronectin-rich basement membrane (Figure 2E). Finally, at day 9, the myocardium had an extensive array fibronectin-rich structures that had a clear tube-like morphology and were highly suggestive of a capillary plexus (Figure 2F). To fully confirm that fibronectin-rich structures are capillaries, future studies will co-stain for endothelial-specific markers such as VE-cadherin or CD31. High magnification images overlaying the fibronectin with the actin and nuclei showed that the fibronectin at day 5 was clearly organized between cardiomyocytes and that over time became thicker until tubes were visible at day 9 (Figure 2G–I). Finally, 3D renderings of the fibronectin confirmed that the structures formed by day 9 were tubes with a fibronectin-rich perimeter and were roughly 10 µm in diameter spaced 10 µm apart, similar to that observed for capillaries in neonatal human hearts [16]. These results show that fibronectin plays a role in capillary formation and that cardiomyocyte alignment occurs prior to vascularization.

### 3.2. Laminin and Fibronectin, but not Collagen Type IV Are Found in the Left Ventricle Myocardium at Day 5

**Figure 3:**
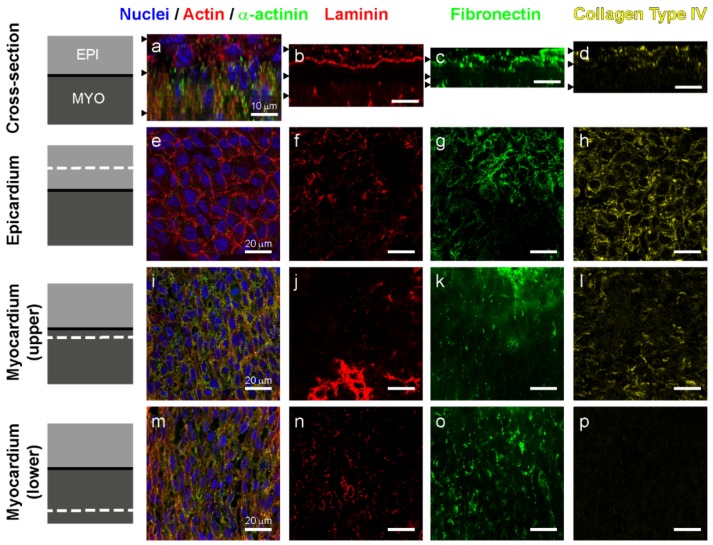
Structure and localization of fibronectin, laminin, and collagen type IV in the epicardium and compacted myocardium of the left ventricle at day 5. (**a**–**d**) Cross-sections of 3D z-stacks with black arrowheads marking the location of each slice for (**e**–**h**), (**i**–**l**), and (**m**–**p**) from top to bottom. (**a**) Cross-section of a 3D confocal z-stack showing the epicardium (α-actinin negative) and the underlying myocardium (α-actinin positive–green). (**b**–**d**) Cross-sections of 3D images showing respectively laminin, fibronectin, and collagen type IV. (**e**) A single image of the epicardium showing polygonal shaped cells cortical actin at the borders of the epithelial cells. (**f**–**h**) The epicardium has laminin, fibronectin, and collagen type IV throughout. (**i**) In the upper myocardium, just below the epicardium, aligned cardiomyocytes are visible and express α-actinin. (**j**–**l**) The laminin and collagen type IV are decreased compared to the epicardium, while fibronectin is also reduced but fibers are clearly aligned in direction of the cardioymocytes. Note that laminin staining in (**j**) is due to an artifact of the Z-section including a region of basement membrane. (**m**) In the deeper myocardium, just above the trabeculations, aligned cardiomyocytes are visible as well as some empty spaces. (**n**–**p**) The laminin in this region is further decreased but the collagen type IV is entirely absent, and the fibronectin actually increases and is aligned. Scale bars are 10 µm for (**a**–**d**) and 20 µm for (**e**–**p**).

As a next step, we focused on the ECM associated with the initial uniaxial alignment of the myocardium that occurs prior to capillary formation, which is why we selected day 5 (stage HH 28) hearts for more detailed analysis. Using confocal microscopy, we obtained 3D images from the epicardium through the compact outer layer of the myocardium. Cross-sections created by re-slicing the Z-stack revealed that there was a sharp border between the epicardium and the myocardium, marked by α-actinin staining (Figure 3a), and a well-defined basement membrane, which was rich in laminin (Figure 3b) and also contained fibronectin (Figure 3c) and collagen type IV (Figure 3d). Cells in the epicardium had a cortical actin cytoskeleton with continuous staining at the cell-cell borders characteristic of the epithelium (Figure 3e) and were surrounded by an ECM of laminin (Figure 3f), fibronectin (Figure 3g) and collagen type IV (Figure 3h). Just below the epicardium, the myocardium is the densest (Figure 3i), with little laminin (Figure 3j), sparse fibronectin fibers that are aligned with the main orientation of the cardiomyocytes (Figure 3k), and traces of collagen type IV (Figure 3l). Deeper in the myocardium, there were intercellular spaces in the tissue (Figure 3m), which had staining for laminin (Figure 3n) and fibronectin (Figure 3o), but not collagen type IV (Figure 3p). The empty spaces in this deeper myocardium were due to starting to transition to the trabeculated myocardium on the endocardial surface that continued throughout the ventricle at this time point. The differences in the laminin, fibronectin, and collagen type IV between epicardium and myocardium was evident without needing to rely on the α-actinin staining of the cardiomyocytes. The absence of collagen type IV in the myocardium at this early stage is not surprising because though it is an essential component of the fetal cardiac ECM (with one study measuring its contribution as 8% of the total weight of ECM [12]), collagen IV only appears after laminin, as a late marker of maturation of the coronary capillaries [17].

### 3.3. Fibronectin and Laminin are Colocalized in Intercellular Spaces in the Avascular Myocardium

**Figure 4:**
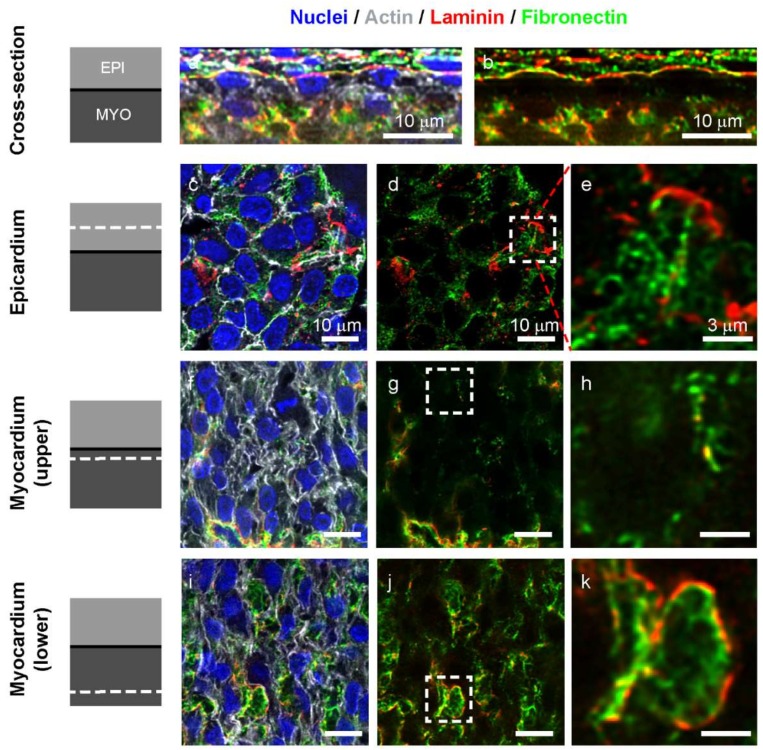
Colocalization of fibronectin and laminin in the myocardium of the left ventricle at day 5. (**a**,**b**) Cross-sections of a single z-stack of the myocardium of the ventral left ventricle at day 5 stained for nuclei (blue), F-actin (grey), laminin (red), and fibronectin (green). The basement membrane separating the epicardium (top) from the myocardium (below) is well defined and rich in laminin. (**c**,**d**) Image of a single slice of the z-stack in the epicardium showing epicardial cells lying on a mat of fibronectin fibers with sheet-like laminin. (**e**) Zoomed in image of the area marked by dashed white square in (**d**) revealing the interconnected fibronectin and laminin in the epicardium. (**f**,**g**,**h**) In the upper and compacted myocardium, only sparse fibronectin fibers, dotted with punctate laminin are found. (**i**,**j**) Deeper in the myocardium, acellular cavities are found with no F-actin signal but rich in ECM. (**k**) These intercellular spaces are covered by laminin and filled with fibronectin fibers. Scale bars are 10 µm for (**a**,**b**,**c**,**d**,**f**,**g**,**i**,**j**) and 3 µm for (**e**,**h**,**k**).

To better understand the ECM in the myocardium at the day 5 stage, we looked at the colocalization of fibronectin and laminin in greater detail to gain insight into these two essential developmental proteins. The overall thickness of compacted epicardium and myocardium was 20–50 µm. Cross-sections of 3D images showed that cardiomyocytes with aligned actin cytoskeleton were present directly beneath the laminin-rich basement membrane of the epicardium (Figure 4a). At this stage, the basement membrane also stained heavily for fibronectin (Figure 4b). In the epicardium, cells were surrounded by a dense mat of fibronectin fibers with laminin in more of a sheet-like structures (Figure 4c). The fibronectin was in a fibrillar network that is seen between and below the epicardial cells and connected with the laminin-rich basement membrane (Figure 4d,e). Just below the basement membrane in the top layer of the myocardium is a layer of cardiomyocytes with very little presence of fibronectin or laminin (Figure 4b,f). What is present are small fibers of fibronectin decorated with punctate dots of laminin (Figure 4g,h). Approximately 10 µm deeper in the myocardium, cardiomyocytes are less aligned and small acellular cavities are visible, composed of laminin-coated perimeters filled with a network of fibronectin fibers.

The intercellular spaces filled with ECM that we observed at this stage have been previously described, and Icardo et al. hypothesized that continuous synthesis of ECM within the myocardium leads to an increase of the hydrostatic pressure and to the formation of these intercellular spaces [18]. Below the compacted myocardium, they might also be signs of the underlying transition to and beginning of the trabeculated myocardium. These intercellular spaces could also be a precursor to the invasion of vascular cells as fibronectin is known to serve as tracks to guide the development of new vessels during coronary vasculogenesis [17]. These ECM-filled acellular cavities were present across samples studied at this timepoint and likely serve multiple roles during cardiac morphogenesis.

### 3.4. Quantitative Analysis of the Fibronectin and Laminin Matrix

**Figure 5:**
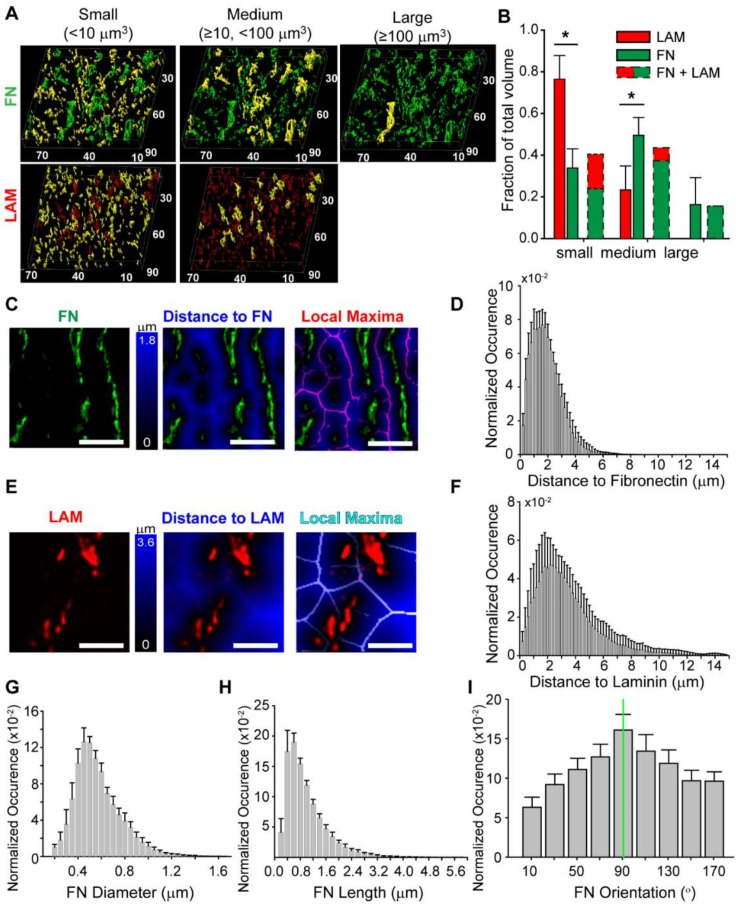
Quantitative analysis of fibronectin and laminin in the myocardium at 5 days. (**A**) ECM structures segmented using Imaris into “surfaces” were sorted into small, medium, or large groups depending on their volume. The ECM structures in each group are highlighted in yellow in 3D representations of fibronectin (green) and laminin (red). Scale is in micrometers. (**B**) Volume distribution of fibronectin and laminin, the dashed bars indicate the relative volume of each component (*, *p* < 0.01 determined by t-test). (**C**) The fibronectin image (green) was processed to find the distance of all points in the image from the fibronectin “surface,” where the value of each voxel is equal to the closest distance of the voxel to a fibronectin “surface.” The local maxima is a 2D image with all points equidistant from fibronectin “surfaces” highlighted in purple. Scale bars are 4 µm. (**D**) Histogram of distance to fibronectin showing mean ± standard deviation for each bin across all samples (*n* = 11). (**E**)The laminin image (red) was processed to find the distance of all points in the image from the laminin “surface,” where the value of each voxel is equal to the closest distance of the voxel to a laminin “surface.” The local maxima is a 2D image with all points equidistant from laminin “surfaces” highlighted in light blue. Scale bars are 4 µm. (**F**) Histograms of distance to laminin showing mean ± standard deviation for each bin across all samples (*n* = 6). (**G**–**I**) Histograms of fibronectin fiber diameter, length, and orientation angle. fibronectin fibers were aligned in the main direction of myofiber orientation (green line) at 90°.

To further characterize the ECM, we focused on the myocardium 5–10 µm below the epicardium and used Imaris to segment the fibronectin and laminin images and extract structural parameters. First, we created “surfaces” (3D objects created by Imaris based on a chosen threshold and described by a set of statistics such as volume, orientation, channel intensity, etc.) and sorted these according to their volume into small (<10 µm^3^), medium (≥10 µm^3^ and <100 µm^3^), and large (≥100 µm^3^) categories. In representative z-stacks for each protein, small ECM structures were predominant while large elements were occasionally present in the fibronectin matrix but never in the laminin (Figure 5a). Overall the fibronectin consisted of 34% small, 50% medium, and 16% large structures while the laminin consisted of 77% small and 23% medium structures (Figure 5b).

To determine the spatial organization of the fibronectin and laminin, we calculated the distance of each voxel in the 3D z-stack to the nearest segmented ECM “surface”. For fibronectin, the fibers were generally uniaxially aligned and the maximum distance between “surfaces” was half the interfiber spacing (Figure 5c). Plotting this data as a histogram, we found that the mode for fibronectin was 1.6 µm, and therefore the approximate spacing between fibronectin fibers was 3.2 µm with an asymmetric distribution and positive skew up to ~6 µm (Figure 5d). For laminin, the morphology of the stained structures was not really fibrillar and the distance between “surfaces” analysis produced a more polygonal morphology (Figure 5e). Plotting this data as a histogram, we found that the mode for laminin was 2.2 µm, and therefore the approximate spacing between laminin structures was 4.4 µm with an asymmetric distribution and positive skew up to ~14 µm (Figure 5e). This distribution reflects the decreased amount of laminin compared to fibronectin, and thus greater spacing. One limitation with this measurement is the border effect, primarily affecting the positive skew of the distribution in the right tail of the distribution due to structures that are out of the field of view and further away than can be observed. Despite this, the distance to ECM “surface” provides a basic analysis of the spacing and distribution of ECM structures in the myocardium.

We further characterized the fibronectin matrix because the fibrillar structure enabled more detailed quantification. To do this we created “filaments” (3D objects created by Imaris using a threshold as well as skeletonization to segment fibrous structures) and extracted information on diameter, length, and orientation. The fibronectin fibers were 0.5 ± 0.2 µm in diameter with an average length of 0.9 ± 0.6 µm (Figure 5g,h). It is important to note that in this analysis a fiber s defined as a segment of fibronectin between two intersections, meaning that two long fibers connecting at their middle will be counted as four fibers of half the length. Thus, small fibers counted were typically segments in large fibronectin fibers. To quantify alignment, we examined fibronectin fibers with a length above 1 µm (31% of all fibers) and found that they were aligned in the same direction as the cardiomyocytes in the myocardium (Figure 5i).

### 3.5. Fibronectin Localization at the Whole-Heart Scale

**Figure 6:**
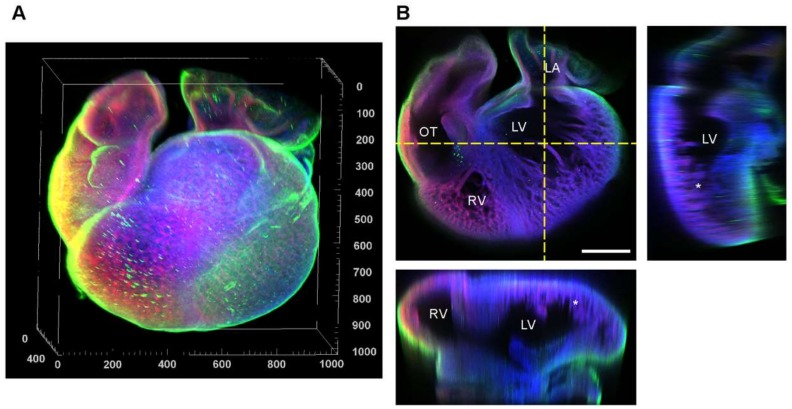
Whole-mount confocal imaging of the day 5 chick heart using refractive index matching with BABB. (**A**) 3D rendering of the heart stained for nuclei (blue), F-actin (red), and fibronectin (green). Scale is in micrometers. (**B**) Three different cross-sections of the heart in the planes marked by yellow doted lines show the different chambers and the trabeculation of the myocardium at this stage (marked by *). Scale bar are 200 µm. RV = right ventricle, LV = left ventricle, OT = outflow track, and LA = left atrium.

Finally, having investigated the organization of fibronectin at high magnification we imaged the entire heart at 5 days to visualize differences throughout the organ. To do this, we cleared the heart using BABB, stained for nuclei, F-actin and fibronectin, and imaged using whole-mount confocal microscopy. The heart has the basic four-chamber structure and the epicardium on the outside stained brightly for fibronectin (Figure 6A). Cross-sections generated from the z-stack revealed that the ventricles were highly trabeculated and that the compact and aligned myocardium that we analyzed in previous figures was only found at the epicardial surface (Figure 6B). From these images, it is apparent that fibronectin ECM is found at much greater relative density in the epicardium, and this is consistent with the results in Figure 3 and Figure 4.

## 4. Discussion

Our results confirm previous studies that have examined the role of fibronectin and other ECM proteins in early cardiac morphogenesis, and also provide some additional insights. It is known that fibronectin, laminin, and collage type IV are important during cardiac morphogenesis by enabling the migration of endothelial and vascular smooth muscle cells [10] and promoting cardiomyocyte proliferation [12,19]. Further, fibronectin is essential during vascular morphogenesis [20,21] and is an early marker of capillary formation [17]. In the early stages of cardiac morphogenesis, the avascular myocardium increases in mass through the formation of trabeculae that are thin enough for diffusion from blood within the ventricles. Vascularization starts with invasion of cells from the sinus venosus over the epicardium and into the myocardium at stage HH27 (day 5) [5,22]. These cells, joined by endocardial cells forming “blood islands” in the myocardium, form a coronary plexus which slowly covers the entire myocardium starting from the dorsal atrioventricular grooves. Then “capillary-like” structures connect to the aorta by stage HH32 (day 7.5), followed by remodeling of the coronary plexus into a network of coronary arteries, veins, and capillaries [23,24,25]. Thus, these studies and our results indicate that microvascularization is associated with a fibronectin-rich ECM.

A key observation of this study is that the cardiomyocytes in the outer most layer of the myocardium, just below the epicardium, are already aligned prior to capillary formation, but continue to mature. As observed at day 5, the cardiomyocytes are uniaxially aligned at this early time point (Figure 2a) and the fibronectin matrix is composed of thin fibers aligned in the same direction (Figure 2d). However, even though alignment is present, the myofibrils appear to be a bit wavy in structure and there is not a visible H-band, indicating that sarcomeres are not completely formed (Figure 2g) [26,27]. At day 7, striations appear in the myofibrils (Figure 2h) and by day 9 the myofibrils are straight, striated, and H-zones are aligned across multiple myofibrils (Figure 2i). This structural maturation of the cardiomyocyte cytoskeleton occurs together with the formation of the capillary-like structures, and unfortunately it is not possible to decouple the role these fibronectin-rich ECM structures may serve as physical guidance cues from other mechanical and cell signaling cues. Our analysis also looks at only a few major ECM components, and studies have shown that there are a wide range of proteins that could have an impact on muscle formation. For example, fibrillin-1 has been shown to play a role in cardiomyocyte proliferation [12], although fibrillin does require fibronectin for assembly [28], suggesting that different ECM components are required to work together and that this changes based on developmental stage.

A lasting question regarding the structural analysis carried out here is how much resolution is needed? The smallest features we can accurately measure with confocal microscopy are on the order of half the wavelength of the light used, which is ~250 nm in the XY focal plane of the objective [29]. The analysis we did on fibronectin filaments in the myocardium at day 5 showed most had diameters of ~500 nm (Figure 5g), however it is possible that these fibers could have been multiple smaller fibers together that could not be resolved. Does the structure of the fibronectin molecule matter? Single fibronectin strands are 2 nm wide, with domains that uncoil under stress to reveal cryptic binding domains [30,31,32]. There is significant evidence that the nanoscale structure of fibronectin in particular plays a key role in regulating diverse cell behaviors [33,34,35] and the same effect is likely to be observed in many other ECM components. Unfortunately, though there are advanced methods to image cell-ECM interaction at the nanoscale using both optical and electron microscopy, it is not yet possible to do so with whole-mount tissues [36]. The cell response to the microenvironment may indeed depend on the nanometer-scale structure of the ECM. Studies of cardiomyocyte interactions with engineered fibronectin surfaces have shown response to both nanometer and micrometer scale features [37,38,39], suggesting both length scales are important.

There are also fundamental limitations to our approach based on the optical methods and image processing techniques used. Measuring structural parameters using immunofluorescent staining and confocal microscopy has several limitations. First, it is difficult to estimate the total amount of protein within the volume of myocardium imaged. Accurate measures of the protein mass require mass spectroscopy. The volume or intensity of the fluorescent signal are poor estimation of the amount of protein as they rely heavily on the quality of staining. Second, the results of the data analysis are dependent in some part on the parameters used in the image segmentation algorithms. We used the same parameters for imaging and processing all samples.

Finally, our results are also of interest beyond cardiac development, such as for developing design parameters for tissue engineering scaffolds. The transformation of the myocardium from a single layer of avascular cardiomyocytes to a highly aligned, multilayered, vascularized tissue is of interest for a wide range of applications. For example, in vitro models of engineered cardiac muscle are growing in importance as disease models and as platforms for drug discovery and toxicity screening [38,39,40,41,42,43]. There are also emerging attempts to engineer 3D cardiac tissue towards in vivo repair and potential organ replacement using decellularization and recellularization and 3D bioprinting [44,45,46,47]. Since aligned and vascularized myocardium forms during embryonic development, this may indeed be the type of ECM scaffold to engineer. As a starting point, our results suggest a scaffold of aligned fibronectin fibers with 0.5 µm diameter and 1 µm length and spaced every 3 µm could work (Figure 5). Thus, our analysis of fibronectin in the chick heart serves to add additional information on how the ECM is organized during development and provide quantitative metrics that can be used to better understand cardiogenesis.
